# Linking What I Say and What I Do: Evidence From Perceived Competition Networks

**DOI:** 10.3389/fpsyg.2022.887510

**Published:** 2022-05-13

**Authors:** Fengwen Chen, Jingwei Xu, Wei Wang, Fangnan Liao, Yineng Guo

**Affiliations:** ^1^School of Economics and Business Administration, Chongqing University, Chongqing, China; ^2^College of Economics and Management, Southwest University, Chongqing, China; ^3^School of Management and Engineering, Nanjing University, Nanjing, China

**Keywords:** perceived competition networks, perceived pressure, corporate risk-taking, peer firms, social interactions

## Abstract

The enterprise network is of great significance in explaining the risk-taking of individual firm. However, some unobservable networks hidden in different firms have long been neglected. Using the text data of the annual reports of China’s listed firms from 2007 to 2018, this paper adopts a textual analysis method to capture the managers’ perceptions of pressure, and build a special kind of hidden inter-firm networks, that is, the perceived competition networks of managers. In addition, this paper discusses the impact of network characteristics on corporate risk-taking behavior. Empirically, there is a positive association between competition strength and corporate risk-taking, as well as the density of perceived competition network. Furthermore, this paper explores the risk-taking behaviors of peer firms in focal firm’s perceived competition network, and finds that the improvement of peer firms’ risk-taking significantly increases the risk bearing level of focal firm, that is, the positive spillover effect of risk-taking behavior among firms in perceived competition networks. Moreover, managers’ personal traits significantly moderate the impact of network characteristics on corporate risk-taking, which is mainly reflected in younger and male managers. Our findings can enrich the literature on social interactions and corporate behaviors, and help firms to improve their understanding of perceptible peer firms.

## Introduction

Corporate risk-taking plays a vital role in the long-term development of firms. A high level of risk-taking can help managers obtain higher investment returns, improve future financial performance, and enhance competitive advantages (Cucculelli and Ermini, 2012). A great quantity of studies have investigated the drivers of corporate risk-taking, most of which focus on the firm-level factors (Bhagat et al., 2015; Mollah et al., 2017). Moreover, some factors related to managers have also been discussed, such as age, gender, educational experience and professional experience (Faccio et al., 2016; Farag and Mallin, 2018). In some industries, market competition can determine the risk-taking of firms, which will be regarded as the external drivers (González et al., 2017; Tongurai and Vithessonthi, 2020). Although existing studies have demonstrated the impact of competition on corporate risk-taking, most of them measure the degree of competition at the industry level, neglecting the role of interactions between different firms in changing managers’ preference, such as Herfindahl-Hirschman Index and Lerner Index.

From the theory of upper echelon, an individual firm can be shaped by the characteristics of its top managers (Durana et al., 2021). As different managers always have distinct personal traits, their cognitions of business environment may vary from person to person, which will lead to the differentiation of decision-making (Elosge et al., 2018). In the light of this view, competition will be perceived by managers, and the managers’ cognitions of competitive environment can measure the degree of competition. According to the industrial organization view, some firms with similar characteristics can be clustered in smaller groups, and there are closer links between such firms in the same group (Zucchini et al., 2019; Changoluisa and Fritsch, 2020). On one hand, the complexity of internal and external environment forces managers to pay more attention to some firms with similar business or product, and then construct a portfolio of competitors (Medhi and Allamraju, 2022). On the other hand, firms often obtain competitive pressure from some firms in similar markets, but neglect the operations and goals of other firms in different markets (Zucchini et al., 2019). More specifically, some managers will feel more pressure from other firms with similar characteristics to perform well, and this interaction may mitigate the agency conflict and managerial slack.

Managers will be influenced by their perceptions of competition when making decisions, especially in some important investments (Sedliacikova et al., 2021). For instance, the decision to enter a new market largely depends on the actions of competitors, which can be regarded as a special kind of pressure (Li et al., 2013). According to the behavioral agency theory, the risk preference of managers is affected by their compensation, which will be tied closely with competitors’ performance (Jayaraman et al., 2021). In other words, managers are willing to prevent some potential losses on their personal wealth by taking few risks, but they may bear more risks facing job termination and eventual bankruptcy (Larraza-Kintana et al., 2007). When new entry threats exist in markets, the relative performance evaluation will force managers to outperform these new competitors in order to maintain their market share (Chen and Liu, 2019). Due to the threats of different competitors, firms need to design new development strategies, such as innovation, price and brand, and this will lead to the high level of risk-taking (Hudakova et al., 2021). In this situation, managers perceived more pressure from their competitors will make more efficient investments in long-term development, and achieve the goal of maximizing profits.

In order to describe the competition environment faced by managers, we construct the perceived competition network for each firm, and capture the closer links between focal firm and its perceptible peer firms. Based on the data of China’s listed firms from 2007 to 2018, the descriptive text of annual reports is used to represent what managers say, and the degree of competition can be measured by the similarity of such text between different firms. In terms of what managers do, we use corporate risk-taking to show managers’ risk preference, and explore the relationship between perceived competition network and risk-taking behavior. Empirically, there is a positive association between the characteristics of perceived competition network and the level of corporate risk-taking, including competition strength and network density. Moreover, we find that the risk-behaviors of perceptible peer firms can promote the risk bearing level of focal firm, indicating that there is a spillover effect of risk-taking behavior in perceived competition network. Furthermore, the personal traits of managers can change the impact of perceived competition network on corporate risk-taking, especially in younger and male managers.

There are some contributions in this paper. First, we capture the managers’ perceptions of competition pressure based on the descriptive text of annual reports, and construct the perceived competition network for each firm. Compared with some existing methods, our method focuses on the semantics of descriptive text in annual reports to represent the ideas of managers, and identify some perceptible peers by using the semantic similarity between different firms (Li et al., 2013; Hoberg and Phillips, 2016). Second, we explore the factors of corporate risk-taking from the perspective of competition network. In the perceived competition networks, the interactions between focal firm and its perceptible peer firms can explain the risk bearing level of focal firm, which represents the risk preference of focal firm’s manager. Third, the personal traits of managers are further demonstrated that the age and gender of managers can change the impact of perceived competition network on corporate risk-taking. We provide the detailed evidence that the young and male managers may perceive more pressure from competitive environment, and they prefer to imitate the behaviors of perceptible peer firms. Our findings can establish where the competition pressure come from, and explain why managers could bear more risks, which will enrich the literature on social interactions and corporate behaviors.

The rest of this paper is structured as follows: Section “Literature Review and Research Hypotheses” introduces relevant theories and puts forward hypotheses. Section “Model Framework” describes the data and methods. The empirical results as well as explanations and discussions are given in Section “The Analysis of Empirical Results.” Section “Conclusion and Recommendations” puts forward the conclusions.

## Literature Review and Research Hypotheses

### Corporate Risk-Taking

Corporate risk-taking can be regarded as the determining factor for the vitality of businesses (Li et al., 2013), and also plays a vital role in strategic management (Hoskisson et al., 2017). The importance of corporate risk-taking can come from both practical and academic fields. Considering the dynamic nature of managerial decision-making, the risk bearing level of different firms is quite different, so that researchers have always been very interested in exploring the factors of corporate risk-taking (Slattery and Ganster, 2002; Langenmayr and Lester, 2018; Connelly et al., 2020). Nowadays, some factors have been widely discussed from three dimensions, including macro environment, organizational factor and manager characteristic.

In terms of the macro factors of corporate risk-taking, the economic and cultural environment can change the development of firms through regional development and industrial policies (Arif and Lee, 2014). From macro-economic environment, the development of capital market has been proved to enhance the risk bearing level of firms in developed countries (Habib and Hasan, 2017). Some social factors, such as individualism (Li and Zahra, 2012) and uncertainty avoidance (Kwok and Tadesse, 2006), can also affect corporate risk-taking. In terms of the organizational factors of corporate risk-taking, some financial indicators are important factors in affecting the risk bearing level of firms (Sedliacikova et al., 2021). Specifically, these indicators, such as market to book value ratio, ownership structure and scale, are positively correlated with corporate risk-taking (Bhagat et al., 2015). In addition, corporate governance is another important factor affecting corporate risk-taking, and reasonable compensation can allow firms to bear higher risks (Bhagat and Bolton, 2008), as well as good governance structure (Mollah et al., 2017). In terms of the personal factors of corporate risk-taking, the impact of manager characteristics on corporate risk-taking has been a hot topic in corporate finance (Desender et al., 2013; Kordsachia, 2021). The agency theory discusses two sources of corporate risk-taking, namely compensation risk and employment risk, which can also influence the risk preference of managers (Larraza-Kintana et al., 2007). However, managers’ risk preference will change with the changes of enterprise performance, which leads to the inaccuracy of predicting the risk bearing level of firms (García-Granero et al., 2015). As an important factor of corporate behaviors, the traits of managers have been proved to be a predictor of corporate risk-taking (Brookman and Thistle, 2009; Orens and Reheul, 2013). Faccio et al. (2016) made an analysis of the relationship between CEO gender and corporate risk-taking, and found that female CEOs can decrease the risk bearing level of firms. Farag and Mallin (2018) demonstrated that the talented CEOs can improve enterprise performance, and promote corporate risk-taking.

It is worth noting that managers will continue to pay attention to the decision-making of relatively important firms, indicating that there may be some interactions between different firms. Therefore, whether the interactions between managers can influence corporate risk-taking has become the main motivation of this paper.

### Perceived Competitive Pressure and Corporate Risk-Taking

The decisions of firms and the perception of rivals’ actions will be gathered in their business strategies (Hambrick and Mason, 1984). Under the pressure from competitive environment, managers will rely on their competitive advantages to adjust their risk-taking (Wiesner et al., 2018). In the light of dynamic competition theory, managers will perceive the actions of competitors and make corresponding reactions (Hsieh and Hyun, 2016; Medhi and Allamraju, 2022). More specifically, when competitors take actions, the competitive pressure will accumulate within the enterprise network if the focal firm remains inactive and does not respond to the actions of its rivals (Zucchini et al., 2019). In this situation, when managers face great competitive pressure, they will increase their willingness to take risks in order to improve the competitiveness and future performance of companies (Durana et al., 2021), and avoid potential losses or bankruptcy (Hu et al., 2021).

#### The Strength of Perceived Competition Network and Corporate Risk-Taking

In the process of social interaction, the behavior of an individual firm may be affected by the behaviors or characteristics of peer firms (Manski, 2000). Based on the idea of social interactions, some hidden links could be found between firms with similar products, as well as other similar dimensions (Eisdorfer et al., 2021). In our method, we use the similarity of perceived competition between different firms to construct the perceived competition network.

The perceived competition network constituted by firms with similar perceived pressure can influence the risk-taking of focal firm through the links. Because the relationship between firms in enterprise network is the foundation of economic links, the structure of perceived competition network may influence the decision making of such firms (Granovetter, 1985). Among enterprise networks, inter-firm network is most representative, the operation of which builds on the trust, common interests and reputation as a result of the mutual interaction between firms, instead of relying on price (Kuhnen, 2009). Existing studies on inter-firm network focus on the real links, and few of them notice the role of hidden links in constructing such network. The perceived competition network is based on the hidden similarity relationship, and describes the degree of competition pressure between focal firm and its peer firms. This similarity can reflect the intensity of managers’ perceived competition pressure, and show the competitive environment faced by focal firm (Augusto and Coelho, 2009; Lee et al., 2015). Firms in more competitive environment will experience faster changes and have more opportunities, but at the same time, they may also face greater uncertainty and greater pressure, which may promote their risk bearing level (Ang, 2008).

In the perceived competition network, this special kind of enterprise network may influence corporate risk-taking in two ways. On the one hand, trust can reduce the managers’ perception of risk and enhance the risk preference of managers. From the perspective of trust construction, individuals are willing to pay more attention to people with similar characteristics, eliminating misunderstandings and enhancing interactions (Sedliacikova et al., 2021). When managers believe that the some people are trustworthy, they will take more risks in imitating their behaviors. In other words, the higher level of trust will reduce the level of perceived risk, resulting in higher risk bearing level. On the other hand, the competition between firms with higher similarity will be more intense, and managers will face greater pressure from competitive environment. In order to avoid job termination and eventual bankruptcy, managers will increase their willingness to take more risks (Hu et al., 2021).

Based on the above arguments, the increase of perceived competition pressure faced by managers can enhance mutual trust, and reduce perceived risk, which may promote corporate risk-taking. Therefore, this paper proposes the following hypothesis:

H1a: There is a positive association between the strength of perceived competition network and corporate risk-taking.

#### The Density of Perceived Competition Network and Corporate Risk-Taking

Network density describes the depth and breadth of nodes, and represents the degree of interconnection between firms in the perceived competition network (Zhang and Guan, 2019). The increase in the density of perceived competition network could increase companies’ risk-taking. On the one hand, focal firm is embedded in its competitive environment, and faces peer firms with similar characteristics. In order to gain competitive advantages, the manager of focal firm need to work more on enterprise performance, and improve profits to outperform peer firms (Jayaraman et al., 2021). Faced with more peer firms in perceived competition network, focal firm will get more motivations to imitate the behaviors of others, which could strengthen its risk bearing level.

On the other hand, the interactions between network nodes make it easier to acquire information from other nodes (Skilton and Bernardes, 2015). The competitive interaction among firms provide a channel for the transmission of knowledge and perception, and help such firms in perceived competition network obtain more resources and information. It is worth noting that corporate risk-taking is regarded as a resource consuming activity with strong resource dependence (Eklund and Mannor, 2021). In this situation, the perceived competition network can help firms to obtain the required resources, and support focal firm or peer firms to make the appropriate reactions to the actions of other firms in this network (Zucchini et al., 2019).

Based on the above arguments, the higher the density of perceived competition network, the easier to obtain more resources or information, and this process will promote the risk management of firms, which will lead to high risk bearing level. Therefore, this paper proposes the following hypothesis:

H1b: There is a positive association between the density of perceived competition network and corporate risk-taking.

### The Risk-Taking of Peer Firms in Perceived Competition Network

Individual behavior will be affected by the behaviors or characteristics of other individuals in the same group (Manski, 2000). Therefore, companies can also be influenced by their peers within the group during the process of decision making (Dougal et al., 2015). In perceived competition network, the links represent the similar characteristics perceived by managers, which can promote mutual communication and social learning between different firms (Kaustia and Rantala, 2015). Managers who perceive the behaviors of peer firms will make corresponding response to such behaviors, as well as corporate risk-taking. Considering the nature of risk-taking behavior, the information about corporate risk-taking is an important factor for the decision making of other firms. Therefore, it is difficult to obtain some valuable information from other firms, which are not in perceived competition network, and this will increase the uncertainty of social and economic environment (Gupta and Misangyi, 2018).

In the process of decision-making, managers will learn and extract information from peer firms, and ultimately make decisions based on the actions of peer firms (Cao et al., 2019). The interactions between focal firm and its peer firms may change the risk preference of focal firm’s manager, which could affect the risk bearing level of focal firm (Seo, 2021). From the perceptions of competition pressure, the manager of focal firm can get more learning motivations from peer firms in perceived competition network, and their decision making will be more and more similar.

Based on the above arguments, the social interactions between focal firm and its peer firms in perceived competition network may change the risk preference of focal firm’s manager in decision-making, which will also affect the risk bearing level of focal firm. Therefore, this paper proposes the following hypothesis:

H2: In the perceived competition network, the risk-taking of peer firms will significantly affect the risk-taking of focal firm.

## Model Framework

### Sample Selection and Data Sources

The research sample in this paper is the listed firms in the Shanghai Stock Exchange and the Shenzhen Stock Exchange, and the sampling period is from 2007 to 2018. Some samples falling into one of the following categories are excluded: (1) samples in the financial sector; (2) samples with missing data in the variables; (3) samples that are listed for less than 5 years; (4) samples whose descriptive text in annual reports cannot be extracted by computers. Ultimately, a total of 15,672 observations are finally obtained. The data employed in this paper is mainly composed of financial data and stock data, both from the China Stock Market & Accounting Research Database (CSMAR) and Chinese Research Data Services Platform Database (CNRDS). In addition, the text data on competition come from annual reports published on the websites of the Shanghai Stock Exchange, the Shenzhen Stock Exchange, and the listed firms. All continuous variables are winsorized at 1% at both tails, which can minimize the influence of extreme values in empirical analysis.

### Perceived Competition Networks by Managers

Boubakri et al. (2013) distinguish network flow models from network architecture models. In network architecture models, the behavior of firm A causes the behavior of firm B to change, then the behavior of firm C, and so forth. The information related to decision-making can be transmitted through close relationships between different firms, and this will also motivate the reactions of firms to respond to the actions of their competitors (Ryou et al., 2022). Based on this idea, the perceived competition networks constructed in this paper can be seen as a network architecture model.

In order to capture the semantics of descriptive text, we adopt a neural network language model to vectorize the text data, which is named as Paragraph2Vec. In this textual analysis method, Distributed Bag of Words model (DBOW) is used to train the vector of descriptive text for each firm, and this model focuses on the overall semantic of text. Furthermore, we use the semantic similarity of descriptive text between different firms to measure the perception of competition pressure faced by managers, which can be computed by the cosine similarity function. Considering the real situation, the top 10 similar firms of focal firm are identified as potential peer firms based on the method proposed by Lee et al. (2015). Finally, if focal firm’s potential peer firms choose focal firm as their potential peer firms, the links between focal firm and such potential peer firms will be retained, and we can get the perceived competition network for this focal firm.

**FIGURE 1:**
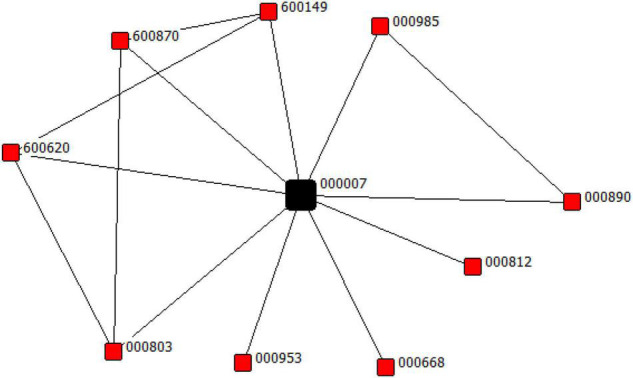
The perceived competition network of focal firm (000007) in 2016.

Figure 1 presents an example of perceived competition network for focal firm 000007 in 2016. As shown in this network, the peer firms of 000007 are these red nodes, including 000029, 000668, 000803, 000812, 000890, 000953, 000985, 600149, 600620, 600870. If 000007 reacts to the action of 000985, 000890 may respond to the action of 000007. Similarly, if 000007 fails to respond to the action of 600149, 000890 would not be prevented from responding to the action of 600149. This suggests that in a similarity network, signals from indirect connections (600149 is indirectly connected to 000890 via 000007) vary with the signals from direct connections. As an ancient proverb goes, the enemy of my enemy is my friend (Davis, 1991). However, what matters to me is what my enemy does. This echoes the finding drawn by Burt that the effective source of stimulus for the focus company is direct connections rather than intermediaries that control the flow of information from the remote part of network (Gimeno, 2004). This is why we focus on the structure of ego-network instead of its position in the entire network.

Based on textual analysis, we identify peer firms with a similar semantic of descriptive text in annual reports. Referring to the parameter settings in Le and Mikolov (2014), we set the dimension of paragraph vector to be 400, and the context window to be 25. Finally, we use the retained links between focal firm and its peer firms to construct the perceived competition network, which are identified by that the manager of focal firm and peer firms will pay more attention to each other.

### The Construction of Variables

#### Corporate Risk-Taking

According to existing studies, higher corporate risk-taking means higher uncertainty about future cash inflows (Faccio et al., 2011; Boubakri et al., 2013), so earnings volatility is often used to measure the level of corporate risk-taking. In this paper, corporate risk-taking is measured by the volatility of return on assets (ROA) in a certain observation period, that is, the ratio of a company’s year-end earnings before interest and tax (EBIT) to the year-end total assets. Specifically, with reference to Faccio et al. (2016), we adjust the ROA of samples in the observation years according to the average of peer firms in perceived competition network for the sake of avoiding the potential influence, thereby obtaining *Adj*_*ROA*_*i*,*t*_ in the observation years. The specific calculation method is shown below:


(1) $$\mathrm{Adj\_}\,R\,O\,A_{i,t}=\frac{E\,B\,I\,T_{i,t}}{A\,s\,s\,e\,t_{i,t}}-\frac{1}{N\,u\,m}\,\sum_{k\in X_{i,t}}^{N\,u\,m}\frac{E\,B\,I\,T_{k,t}}{A\,s\,s\,e\,t_{k,t}}$$

In Equation (1), *Num* is the total number of firms in focal firm i’s perceived competition network in year t, and *X*_*i,t*_ is the set of peer firms in focal firm i’s perceived competition network.

On that basis, this paper uses two methods, denoted as *Risk*_*T*1_*i*,*t*_ and *Risk*_*T*2_*i*,*t*_, to further measure corporate risk-taking. First of all, the standard deviation of *Adj*_*ROA*_*i*,*t*_ for three consecutive years (2005–2007, 2006–2008, 2007–2009…, 2016–2018) is worked out to measure risks taken by listed companies. [In this study, given the fact that the tenure of executives of listed companies in China is generally 3 years, we chose 3 years as an observation period with reference to the research made by Boubakri et al. (2013)]. See Equation (2) for details. Secondly, corporate risk-taking is measured by the difference between the maximum and minimum values of *Adj*_*ROA*_*i*,*t*_ during each observation period (3 years). See Equation (3) for details.


(2) $$\mathrm{Risk\_}T\,1_{i,t}=\sqrt{\frac{1}{T-1}\,\sum_{t=1}^{T}{\left(\mathrm{Adj\_}\,R\,O\,A_{i,t}-\frac{1}{T}\,\sum_{t=1}^{T}\mathrm{Adj\_}\,R\,O\,A_{i,t}\right)}^{2}}|T=3$$

(3) $$\mathrm{Risk\_}\,T\,2_{i,t}=M\,a\,x\,\left(\mathrm{Adj\_}\,R\,O\,A_{i,t}\right)-M\,i\,n\,\left(\mathrm{Adj\_}\,R\,O\,A_{i,t}\right)$$

#### The Characteristics of Perceived Competition Networks

This paper measures the characteristics of perceived competition networks for managers from two dimensions, denoted as *PCN*_*Strength*_*i*,*t*_ and *PCN*_*Desity*_*i*,*t*_. First of all, given that the similarity of firms in annual reports is set as the connection formed between firms in the same perceived competition network, we use the average of perceptible similarity, i.e., competition strength (*PCN*_*Strength*_*i*,*t*_) between focal firm and its perceived peer firms to measure this variable, as shown in Equation (4):


(4) $$\mathrm{PCN}\text{\_}\,S\,t\,r\,e\,n\,g\,t\,h_{i,t}=\frac{\sum_{j\in X_{i,t}}^{k}p\,e\,r\,c\,e\,p\,t\,i\,b\,l\,e\,s\,i\,m\,i\,l\,a\,r\,i\,t\,y_{j,t}}{k},$$

In Equation (4), *PCN*_*Strength*_*i*,*t*_ represents the strength of perceived competition pressure for the manager of focal firm *i* in year *t*; *k* is the number of peers in the perceived competition network of focal firm *i*; *X*_*i,t*_ is the peer firms set of focal firm *i*’s perceived competition network.

In the next place, in order to understand the connection relationship between perceived peer firms in competition networks, this study uses network density (*PCN*_*Desity*_*i*,*t*_) to investigate the cohesion of the perceived competition networks for managers (Zhang and Guan, 2019). Network density is calculated by the actual ties of peer firms divided by the maximum possible ties, as shown in Equation (5):


(5) $$\mathrm{PCN}\text{\_}\,D\,e\,s\,i\,t\,y_{i,t}=\frac{\sum_{j\in X_{i,t}}^{k}t\,h\,e\,a\,c\,t\,u\,a\,l\,n\,u\,m\,b\,e\,r\,o\,f\,t\,i\,e\,s\,a\,m\,o\,n\,g\,p\,e\,e\,r\,s_{i,j,t}}{k\,\left(k-1\right)/2},\;j\in s\,p\,m_{i,t}$$

#### Risk-Taking Behaviors of Peer Firms

This study further examines the influence of peer firms’ risk-taking behaviors in perceived competition network on the risk-taking of focal firm. Consistent with the previous section, the peer firms of focal firm i come from the set of *X*_*i,t*_. On that basis, this study measured the risk-taking of peer firms using Equation (6) and Equation (7):


(6) $$\mathrm{Risk}\text{\_}\,T\,1_{X_{i,t},t}=\frac{\sum_{j\in X_{i,t}}^{P\,e\,e\,r\,N\,u\,m_{i,t}}\mathrm{Risk}\text{\_}\,T\,1_{j,t}}{P\,e\,e\,r\,N\,u\,m_{i,t}}$$

(7) $$\mathrm{Risk}\text{\_}\,T\,2_{X_{i,t},t}=\frac{\sum_{j\in X_{i,t}}^{P\,e\,e\,r\,N\,u\,m_{i,t}}\mathrm{Risk}\text{\_}\,T\,2_{j,t}}{P\,e\,e\,r\,N\,u\,m_{i,t}}$$

Where, *Risk*_*T*1_*X*_*i*,*t*_,*t*_ and *Risk*_*T*2_*X*_*i*,*t*_,*t*_ represent the risk-taking of peer firms in focal firm i’s perceived competition network; *PeerNum*_*i*,*t*_ represents the number of peer firms in *X*_*i,t*_; *Risk*_*T*1_*j*,*t*_ and *Risk*_*T*2_*j*,*t*_ represent the risk-taking of firm *j* in year *t*.

#### Control Variables

Referring to existing research (Farag and Mallin, 2018; Langenmayr and Lester, 2018; Hu et al., 2021), this study controls other factors possibly influencing corporate risk-taking, including firm size (*Size*) that is the natural logarithm of the firm’s total assets, financial leverage (*Lev*) that is the ratio of the firm’s total liabilities to total assets; enterprise age (*Listage*) that is the number of years of the firm’s establishment plus 1; turnover rate (*Turnover*) that is the proportion of stock trading volume in the total number of shares; dual occupancy (*Dual*) that is assigned to 1 if the chairman and CEO of the firm are served by the same person, or otherwise 0; ownership (*Ownership*) that is assigned to 1 if the firm is state owned, or otherwise 0; state-owned holding ratio (*State*) that means the proportion of the state-owned shares of the firm; and equity concentration (*Top3*) that indicates the sum of the proportions of shares held by the firm’s top three shareholders.

The definitions and measurements of variables are shown in Table 1.

**TABLE 1:** The definitions of variables.

Variable	Definition and measurement
*Risk_T*1	Corporate risk-taking. See Equation (2) for its specific calculation.
*Risk_T*2	Corporate risk-taking. See Equation (3) for its specific calculation.
*PCN_Strength*	Strength of perceived competition network faced by managers. See Equations (4) for its specific calculation.
*PCN_Density*	Density of perceived competition network faced by managers. See Equation (5) for its specific calculation.
*Risk*_*T*1_*X*_*i*,*t*_,*t*_	Risk-taking of peer firms that is calculated based on *Risk_T*1. See Equation (6) for its specific calculation.
*Risk*_*T*2_*X*_*i*,*t*_,*t*_	Risk-taking of peer firms that is calculated based on *Risk_T*2. See Equation (7) for its specific calculation.
*Top3*	Ownership concentration. Sum the top three shareholders’ shareholding ratio.
*Ownership*	Nature of ownership. Set state-owned listed enterprises to be 1, or otherwise 0.
*Dual*	Dual occupancy. For the firm whose chairman and CEO are served by the same one person, set it to be 1, or otherwise 0.
*Size*	Firm size, equal to the natural logarithm of the firm’s total assets.
*Lev*	Financial leverage, representing a firm’s liabilities level, equal to the ratio of the firm’s total liabilities to total assets
*Listage*	Enterprise age, equal to the natural logarithm of the number of years of a firm’s establishment plus 1.
*Turnover*	Turnover rate, equal to the proportion of stock trading volume in the total number of shares.

Risk-taking of peer firms that is calculated based on *Risk_T*1. See Equation (7) for its specific calculation

### The Construction of Empirical Models

In order to verify the hypotheses proposed in Section “Literature Review and Research Hypotheses,” the following regression models are constructed. *Risk*_*Tn*_*i*,*t*_ includes the two corporate risk-taking indicators, *Risk*_*T*1_*i*,*t*_ and *Risk*_*T*2_*i*,*t*_ and *PCN_X* represents the characteristics of the similarity network perceived by managers. Ultimately, *PCN*_*X*_*i*,*t*_ is measured from these two dimensions, denoted as *PCN_Strength* and *PCN_Density*, respectively. Equation (8) is used to test H1a and H1b: The association between perceived competition network and corporate risk-taking.


(8) $$\mathrm{Risk}\text{\_}\,T\,n_{i,t}=\alpha_{0}+\alpha_{1}\,\mathrm{PCN}\text{\_}\,X_{i,t}+\alpha_{3}\,C\,o\,n\,t\,r\,o\,l_{i,t}+Y\,E\,A\,R+I\,N\,D+\varepsilon_{i,t}$$

In Equation (9), we further examine the relationship between the risk-taking of peer firms (*Risk*_*Tn*_*X*_*i*,*t*_,*t*_) and the risk-taking of focal firm (*Risk*_*Tn*_*i*,*t*_) in the perceived competition network, in a bid to verify H2.


(9) $$\mathrm{Risk}\text{\_}\,T\,n_{i,t}=\alpha_{0}+\alpha_{1}\,\mathrm{Risk}\text{\_}\,T\,n_{X_{i,t},t}+\alpha_{3}\,C\,o\,n\,t\,r\,o\,l_{i,t}+Y\,E\,A\,R+I\,N\,D+\varepsilon_{i,t}$$

Where, *Risk*_*Tn*_*X*_*i*,*t*_,*t*_ represents the average risk-taking of peer firms in focal firm i’s perceived competition network including *Risk*_*T*1_*X*_*i*,*t*_,*t*_ and *Risk*_*T*2_*X*_*i*,*t*_,*t*_.

## The Analysis of Empirical Results

### Descriptive Statistics

Table 2 presents the descriptive statistical results of variables in this study. The results show that the maximum and minimum of corporate risk-taking *Risk*_*T*1_*i*,*t*_ (*Risk*_*T*2_*i*,*t*_) are 1.0204 (1.9472) and 0.0028 (0.0053), respectively, suggesting that during the sampling period, China’s listed companies had quite different levels of risk-taking and there were obvious differences in the risk-taking decisions of managers. These findings are basically consistent with existing research results. The mean value of *Risk*_*T*1_*i*,*t*_ (*Risk*_*T*2_*i*,*t*_) is 0.0638 (0.1205), with a median of 0.0328 (0.0622) and a standard deviation of 0.1269 (0.2388). The distribution of corporate risk-taking is relatively scattered. The mean of *PCN_Strength* is 0.2872, with minimum and maximum of 0.0000 and 0.3848, respectively, and a standard deviation of 0.0618. The mean value of *PCN_Density* is 0.0916, with minimum and maximum of 0.0000 and 0.6000, respectively, and a standard deviation of 0.0357. These two network indicators suggest that the perceived competition faced by managers is relatively concentrated, whereas, in terms of network density, peer firms have significant differences in relational decision-making. The mean of *Risk*_*T*1_*X*_*i*,*t*_,*t*_ (*Risk*_*T*2_*X*_*i*,*t*_,*t*_) is 0.0821 (0.1516), with minimum and maximum of 0.0000 (0.0000) and 1.4041 (2.5342), respectively, suggesting significantly different risk-taking levels of peer firms. For control variables, the mean of *Ownership* is 0.6508, indicating that about 65.08% of research samples are state-owned enterprises. The mean of *Size* is 22.2034, which is very close to its median.

**TABLE 2:** The descriptive statistics of variables.

Variables	*N*	Mean	Median	Std	Min	Max
*Risk_T*1	12713	0.0638	0.0328	0.1269	0.0028	1.0204
*Risk_T*2	12713	0.1205	0.0622	0.2388	0.0053	1.9472
*PCN_Strength*	12713	0.2872	0.2918	0.0618	0.0000	0.3848
*PCN_Density*	12713	0.0916	0.0357	0.1284	0.0000	0.6000
*Risk*_*T*1_*X*_*i*,*t*_,*t*_	12713	0.0821	0.0386	0.1858	0.0000	1.4041
*Risk*_*T*2_*X*_*i*,*t*_,*t*_	12713	0.1516	0.0733	0.3335	0.0000	2.5342
*Ownership*	12713	0.6508	1.0000	0.4767	0.0000	1.0000
*Dual*	12713	0.1414	0.0000	0.3484	0.0000	1.0000
*Size*	12713	22.2034	22.0900	1.3991	18.9644	26.0709
*Turnover*	12713	4.2826	3.5936	2.8414	0.3899	13.7451
*Lev*	12713	0.5302	0.5346	0.2105	0.0856	1.1575
*Top3*	12713	0.1670	0.1329	0.1265	0.0097	0.5924
*Listage*	12713	2.7542	2.7726	0.3124	1.7918	3.3322

Table 3 presents the Pearson and Spearman coefficient of correlation. The correlation coefficient of risk-taking indicators (*Risk_T*1 and *Risk_T*2) is 0.999 (0.999), and it is significant at the level of 1%, indicating that two risk-taking indicators are highly consistent. Similarly, the correlation coefficient of *Risk*_*T*1_*X*_*i*,*t*_,*t*_ and *Risk*_*T*2_*X*_*i*,*t*_,*t*_ is 0.999 (0.999), indicating that the two risk-taking indicators are closely associated. The correlation coefficients of network variables (*PCN_Strength* and *PCN_Density*) and risk-taking variables (*Risk*_*T*_1*X*_*i*,*t*_,*t*_, *Risk*_*T*2_*X*_*i*,*t*_,*t*_, *Risk*_*T*1_*i*,*t*_ and *Risk*_*T*2_*i*,*t*_) are positive, and passed the significance testing at the 1% level and 10% level. The absolute correlation coefficients between the control variables and independent variables or dependent variables are less than 0.5, indicating that there is no collinearity problem in empirical models.

**TABLE 3:** The correlation coefficient of variables.

	*Risk_T*1	*Risk_T*2	*PCN_Strength*	*PCN_Density*	Risk_T1_X_i,t_,t_	Risk_T2_X_i,t_,t_	*Ownership*
*Risk_T*1	1	0.999***	0.031***	−0.131***	0.284***	0.285***	−0.112***
*Risk_T*2	0.999***	1	0.031***	−0.131***	0.285***	0.286***	−0.112***
*PCN_Strength*	0.057***	0.057***	1	0.385***	0.233***	0.233***	0.027***
*PCN_Density*	0.015*	0.015*	0.264***	1	0.043***	0.042***	0.057***
*Risk*_*T*1_*X*_*i*,*t*_,*t*_	0.392***	0.392***	0.145***	0.033***	1	0.999***	−0.078***
*Risk*_*T*2_*X*_*i*,*t*_,*t*_	0.395***	0.395***	0.148***	0.033***	0.999***	1	−0.079***
*Ownership*	−0.093***	−0.094***	0.002	0.077***	−0.067***	−0.068***	1
*Dual*	0.038***	0.038***	−0.012	−0.022**	0.021**	0.022**	−0.178***
*Size*	−0.251***	−0.252***	−0.005	0.163***	−0.186***	−0.189***	0.235***
*Turnover*	0.014	0.014	−0.028***	−0.086***	−0.032***	−0.031***	−0.064***
*Lev*	0.111***	0.112***	0.019**	0.019**	0.056***	0.057***	0.069***
*Top3*	−0.110***	−0.110***	0.037***	0.120***	−0.070***	−0.072***	0.237***
*Listage*	−0.003	−0.004	−0.013	−0.003	0.003	0.003	−0.142***
	*Dual*	*Size*	*Turnover*	*Lev*	*Top3*	*Listage*	
*Risk_T*1	0.055***	−0.330***	0.127***	−0.029***	−0.155***	−0.073***	
*Risk_T*2	0.056***	−0.330***	0.126***	−0.029***	−0.154***	−0.072***	
*PCN_Strength*	−0.034***	−0.093***	0.044***	0.043***	0.046***	−0.190***	
*PCN_Density*	−0.016*	0.172***	−0.115***	0.027***	0.116***	0.063***	
*Risk*_*T*1_*X*_*i*,*t*_,*t*_	0.016*	−0.236***	0.056***	−0.059***	−0.101***	−0.040***	
*Risk*_*T*2_*X*_*i*,*t*_,*t*_	0.017*	−0.238***	0.057***	−0.060***	−0.102***	−0.041***	
*Ownership*	−0.178***	0.217***	−0.071***	0.079***	0.272***	−0.141***	
*Dual*	1	−0.094***	0.041***	−0.024***	−0.129***	0.058***	
*Size*	−0.095***	1	−0.343***	0.265***	0.344***	0.037***	
*Turnover*	0.036***	−0.306***	1	−0.043***	−0.401***	0.045***	
*Lev*	−0.017*	0.211***	−0.040***	1	0.031***	0.027***	
*Top3*	−0.114***	0.376***	−0.382***	0.013	1	−0.241***	
*Listage*	0.060***	−0.017*	0.061***	0.030***	−0.241***	1	

***, **, and * represent passing the test at the significance levels of 1%, 5%, and 10%.

### Baseline Test

#### Perceived Competition Networks and Corporate Risk-Taking

To explore the factors of corporate risk-taking, this paper adopts the empirical model constructed by Equation (8) to test the association between perceived competition networks and corporate risk-taking. Firstly, this paper explores the impact of the strength of perceived competition networks on corporate risk-taking. Then, the impact of the density of perceived competition networks on corporate risk-taking is tested by the same regression method. The baseline results are reported in Table 4.

**TABLE 4:** The impact of perceived competition networks on corporate risk-taking.

Variables	Risk_T1_i,t_					Risk_T2_i,t_		
	(1)	(2)	(3)	(4)	(5)	(6)	(7)	(8)
*PCN_Strength*	0.1249***	0.0997***			0.2354***	0.1877***		
	(4.32)	(4.03)			(4.30)	(4.00)		
*PCN_Density*			0.0411**	0.0552***			0.0777***	0.1039***
			(2.34)	(3.44)			(2.34)	(3.42)
*Ownership*		−0.0106**		−0.0109**		−0.0202**		−0.0208**
		(−2.49)		(−2.56)		(−2.51)		(−2.57)
*Dual*		0.0031		0.0029		0.0058		0.0055
		(0.60)		(0.57)		(0.61)		(0.58)
*Size*		−0.0282***		−0.0287***		−0.0531***		−0.0540***
		(−11.03)		(−10.92)		(−10.98)		(−10.87)
*Turnover*		−0.0052***		−0.0052***		−0.0098***		−0.0098***
		(−6.73)		(−6.75)		(−6.74)		(−6.76)
*Lev*		0.1119***		0.1112***		0.2130***		0.2117***
		(6.72)		(6.74)		(6.75)		(6.77)
*Top3*		−0.0391***		−0.0398***		−0.0742***		−0.0756***
		(−2.64)		(−2.68)		(−2.67)		(−2.70)
*Listage*		−0.0078		−0.0066		−0.0152		−0.0129
		(−1.05)		(−0.89)		(−1.09)		(−0.93)
*Constant*	0.0394***	0.6508***	0.0718***	0.6822***	0.0749***	1.2277***	0.1359***	1.2870***
	(3.49)	(11.78)	(8.57)	(11.50)	(3.53)	(11.73)	(8.76)	(11.45)
Observations	12713	12713	12713	12713	12713	12713	12713	12713
Industry fixed	Yes	Yes	Yes	Yes	Yes	Yes	Yes	Yes
Year fixed	Yes	Yes	Yes	Yes	Yes	Yes	Yes	Yes
*R* ^2^	0.0257	0.1187	0.0236	0.1191	0.0262	0.1200	0.0241	0.1205

*** and ** represent passing the test at the significance levels of 1% and 5% respectively; the t-value has been robustly corrected during the statistical test.

Table 4 presents the results of regression testing on whether perceived competition networks can influence corporate risk-taking. Columns (1)-(2) illustrate that the coefficients between *PCN_Strength* and *Risk*_*T*1_*i*,*t*_ are 0.1249 and 0.0997, significant at the 1% level. Columns (3)-(4) illustrate that the coefficients between *PCN_Density* and *Risk*_*T*1_*i*,*t*_ are 0.0411 and 0.0552, significant at the 5% and 1% level. Columns (5)-(6) illustrate that the coefficients between *PCN_Strength* and *Risk*_*T*2_*i*,*t*_ are 0.2354 and 0.1877, significant at the 1% level. Columns (7)-(8) illustrate that the coefficients between *PCN_Density* and *Risk*_*T*2_*i*,*t*_ are 0.0777 and 0.1039, significant at the 5% and 1% level. These results suggest that corporate risk-taking increases as the intensity of perceived pressure faced by managers, as well as the density of competition network, thereby proving H1a and H1b. In terms of control variables, *Ownership*, *Size*, *Turnover*, and *Top3* are found negatively correlated with corporate risk-taking, while *Lev* is positively correlated with corporate risk-taking, which supports the existing research findings (Faccio et al., 2011, 2016). High perceived pressure of managers from other firms’ annual reports can force the focal firm’s manager to make great efforts to improve the ability of risk management. At the same time, the more similar with peer firms, the higher competition faced by focal firms. In this situation, focal firms need rely on some risk-taking activities to enhance their competitiveness.

#### Risk-Taking of Peer Firms in Perceived Competition Networks

Based on the close relationship among firms in perceived competition network, the behaviors of peer firms can motivate focal firm to imitate these behaviors, which can help focal firm to obtain unique information and competitive advantage (Lieberman and Asaba, 2006). In our method, each focal firm will face the unique peer firms based on the construction of perceived competition network, and this can resolve the reflection problem in peer effect (Kelchtermans et al., 2020). In order to further verify H2, the average risk-taking of peer firms (*Risk*_*T*1_*X*_*i*,*t*_,*t*_ and *Risk*_*T*2_*X*_*i*,*t*_,*t*_) is introduced into the empirical model constructed by Equation (9). The regression results of peers’ risk-taking are reported in Table 5.

**TABLE 5:** Risk-taking of peer firms and corporate risk-taking.

Variables	Risk_T1_i,t_		Risk_T2_i,t_	
	(1)	(2)	(3)	(4)
*Risk*_*T*1_*X*_*i*,*t*_,*t*_	0.2636***	0.2288***		
	(10.59)	(10.17)		
*Risk*_*T*2_*X*_*i*,*t*_,*t*_			0.2784***	0.2414***
			(10.58)	(10.15)
*Ownership*		−0.0086**		−0.0162**
		(−2.29)		(−2.29)
*Dual*		0.0032		0.0061
		(0.70)		(0.72)
*Size*		−0.0213***		−0.0399***
		(−10.97)		(−10.93)
*Turnover*		−0.0038***		−0.0072***
		(−5.97)		(−5.99)
*Lev*		0.0912***		0.1732***
		(6.73)		(6.76)
*Top3*		−0.0304**		−0.0577**
		(−2.29)		(−2.32)
*Listage*		−0.0050		−0.0100
		(−0.77)		(−0.82)
*Constant*	0.0516***	0.5055***	0.0957***	0.9474***
	(7.19)	(11.28)	(7.17)	(11.22)
Observations	12713	12713	12713	12713
Industry fixed	Yes	Yes	Yes	Yes
Year fixed	Yes	Yes	Yes	Yes
*R* ^2^	0.1673	0.2206	0.1699	0.2232

*** and ** represent passing the test at the significance levels of 1% and 5% respectively; the t-value has been robustly corrected during the statistical test.

In Table 5, Columns (1)-(2) show that the coefficients between the average risk-taking of peer firms (*Risk*_*T*1_*X*_*i*,*t*_,*t*_) and the risk-taking of focal firm (*Risk*_*T*1_*i*,*t*_) are 0.2636 and 0.2288, significant at the 1% level. Columns (3)-(4) show that the coefficients between the average risk-taking of peer firms (*Risk*_*T*2_*X*_*i*,*t*_,*t*_) and the risk-taking of focal firm (*Risk*_*T*2_*i*,*t*_) are 0.2784 and 0.2414, significant at the 1% level. These results indicate that there is a significant spillover effect in corporate risk-taking in perceived competition network. The occurrence of this phenomenon can demonstrate that the imitation behavior can help focal firm to maintain the dominant position in the product market and obtain advantage information from peer firms in perceived competition network. There is an obvious peer effect in corporate risk-taking for different firms in perceived competition network, thereby proving H2.

### Further Test

#### The Role of Managers’ Age in Perceived Competition Networks

The results of baseline test demonstrate that perceived competition networks have a significant positive impact on corporate risk-taking, suggesting that after perceiving competition pressure from peer firms, managers will tend to make decisions with higher risk. Furthermore, managers’ personal traits may also influence their risk preferences. The personal traits can reflect the values of managers, and influence the choice of business strategies and corporate policies, as well as changing the level of risk taking (Kini and Williams, 2012). When managers face external competition pressure, old managers tend to make conservative decisions, such as following industry standards or historical experience (Herrmann and Datta, 2006). However, young managers are more aggressive and decisive, and will be more willing to try risky decisions to prove their personal abilities (Jenter and Lewellen, 2015). In this situation, the research samples are divided into two subsamples, including the group of young managers and the group of old managers. If the age of manager is younger than the average age of managers in research sample, this manager will be classified in the group of young managers, otherwise this manager will be classified in the group of old managers. This paper still uses the empirical model constructed by Equation (8), and the regression results are reported in Table 6.

**TABLE 6:** Results of young managers and old managers.

Variables	Risk_T1_i,t_				Risk_T2_i,t_			
	(1) young	(2) old	(3) young	(4) old	(5) young	(6) old	(7) young	(8) old
	0.1550***	0.0454*			0.2928***	0.0846*		
*PCN_Strength*								
	(4.24)	(1.77)			(4.21)	(1.75)		
			0.0710***	0.0397**			0.1346***	0.0740**
*PCN_Density*								
			(3.15)	(2.20)			(3.15)	(2.18)
	−0.0095*	−0.0126**	−0.0100*	−0.0128**	−0.0182*	−0.0238**	−0.0191*	−0.0242**
*Ownership*								
	(−1.79)	(−2.18)	(−1.86)	(−2.22)	(−1.81)	(−2.18)	(−1.89)	(−2.23)
	0.0030	0.0033	0.0027	0.0032	0.0053	0.0069	0.0049	0.0067
*Dual*								
	(0.43)	(0.52)	(0.40)	(0.50)	(0.40)	(0.57)	(0.37)	(0.55)
	−0.0341***	−0.0226***	−0.0348***	−0.0230***	−0.0645***	−0.0425***	−0.0657***	−0.0431***
*Size*								
	(−9.46)	(−8.24)	(−9.38)	(−8.18)	(−9.40)	(−8.24)	(−9.32)	(−8.17)
	−0.0061***	−0.0037***	−0.0060***	−0.0037***	−0.0115***	−0.0070***	−0.0114***	−0.0069***
*Turnover*								
	(−6.23)	(−3.88)	(−6.21)	(−3.89)	(−6.25)	(−3.92)	(−6.22)	(−3.93)
	0.1192***	0.0988***	0.1188***	0.0979***	0.2283***	0.1860***	0.2274***	0.1844***
*Lev*								
	(5.25)	(4.84)	(5.28)	(4.85)	(5.28)	(4.85)	(5.31)	(4.86)
	−0.0476**	−0.0300**	−0.0470**	−0.0313**	−0.0914**	−0.0563**	−0.0902**	−0.0586**
*Top3*								
	(−2.12)	(−2.01)	(−2.09)	(−2.07)	(−2.16)	(−2.02)	(−2.12)	(−2.07)
	−0.0031	−0.0103	−0.0007	−0.0097	−0.0065	−0.0195	−0.0021	−0.0182
*Listage*								
	(−0.28)	(−1.32)	(−0.07)	(−1.24)	(−0.31)	(−1.34)	(−0.10)	(−1.27)
	0.7512***	0.5535***	0.7977***	0.5687***	1.4196***	1.0407***	1.5075***	1.0691***
*Constant*								
	(9.52)	(9.58)	(9.44)	(9.37)	(9.49)	(9.59)	(9.40)	(9.36)
Observations	6376	6337	6376	6337	6376	6337	6376	6337
Industry	Yes	Yes	Yes	Yes	Yes	Yes	Yes	Yes
Year	Yes	Yes	Yes	Yes	Yes	Yes	Yes	Yes
*R* ^2^	0.1324	0.1081	0.1316	0.1093	0.1336	0.1096	0.1329	0.1107

***, **, and * represent passing the test at the significance levels of 1%, 5%, and 10%, respectively; the t-value has been robustly corrected during the statistical test.

In Table 6, Columns (1) and (2) illustrate that when the age of manager is younger than the average age of managers in research samples, the coefficient between *PCN_Strength* and *Risk*_*T*1_*i*,*t*_ is 0.1550, significant at the 1% level; when a manager’s age is older than the average age of managers in research samples, the coefficient between *PCN_Strength* and *Risk*_*T*1_*i*,*t*_ is 0.0454, significant at the 10% level. Columns (3) and (4) illustrate that when the age of manager is younger than the average age of managers in research samples, the coefficient between *PCN*_*Density* and *Risk*_*T*1_*i*,*t*_ is 0.0710, significant at the 1% level; when a manager’s age is older than the average age of managers in research samples, the coefficient between *PCN*_*Density* and *Risk*_*T*1_*i*,*t*_ is 0.0397, significant at the 5%level. When *Risk*_*T*2_*i*,*t*_ is deployed to measure corporate risk-taking, the regression results are consistent with the analysis of *Risk*_*T*1_*i*,*t*_. It can be found that the influence of perceived competition network on the group of young managers is stronger than that of old managers, indicating this personal trait can change the risk preference of managers.

#### The Role of Managers’ Gender in Perceived Competition Networks

Due to the differences in physiological characteristics and information processing methods, managers of different genders have great differences in risk attitudes (Roberts and Mroczek, 2008). Studies have shown that female managers tend to be more cautious and prudent than their male counterparts, while male managers have greater confidence in dealing with high-pressure problems, so that they have a higher risk appetite (Huang and Kisgen, 2013). In this situation, the research samples are divided into two subsamples, including the group of male managers and the group of female managers. This paper still uses the empirical model constructed by Equation (8), and the regression results are reported in Table 7.

**TABLE 7:** Results of male managers and female managers.

Variables	Risk_T1_i,t_				Risk_T2_i,t_			
	(1) male	(2) female	(3) male	(4) female	(5) male	(6) female	(7) male	(8) female
	0.0865***	0.1153			0.1622***	0.2108		
*PCN_Strength*								
	(3.11)	(1.15)			(3.07)	(1.12)		
			0.0505***	0.0011			0.0946***	−0.0007
*PCN_Density*								
			(2.85)	(0.03)			(2.83)	(−0.01)
	−0.0104**	0.0040	−0.0106**	0.0042	−0.0197**	0.0073	−0.0201**	0.0078
*Ownership*								
	(−2.08)	(0.20)	(−2.13)	(0.22)	(−2.09)	(0.20)	(−2.14)	(0.21)
	0.0074	−0.0151	0.0072	−0.0177	0.0138	−0.0306	0.0135	−0.0353
*Dual*								
	(1.22)	(−0.86)	(1.20)	(−0.99)	(1.22)	(−0.92)	(1.20)	(−1.04)
	−0.0273***	−0.0290***	−0.0278***	−0.0291***	−0.0514***	−0.0551***	−0.0523***	−0.0553***
*Size*								
	(−9.40)	(−3.53)	(−9.31)	(−3.54)	(−9.34)	(−3.52)	(−9.25)	(−3.54)
	−0.0048***	−0.0028	−0.0048***	−0.0029	−0.0090***	−0.0052	−0.0090***	−0.0053
*Turnover*								
	(−5.89)	(−1.04)	(−5.91)	(−1.05)	(−5.91)	(−1.02)	(−5.92)	(−1.02)
	0.1077***	0.2032***	0.1072***	0.2011***	0.2045***	0.3835***	0.2035***	0.3795***
*Lev*								
	(5.58)	(3.32)	(5.60)	(3.36)	(5.59)	(3.29)	(5.61)	(3.33)
	−0.0335*	−0.0958	−0.0347*	−0.0946	−0.0640*	−0.1798	−0.0662**	−0.1773
*Top3*								
	(−1.91)	(−1.26)	(−1.96)	(−1.23)	(−1.93)	(−1.24)	(−1.99)	(−1.22)
	−0.0017	−0.0572	−0.0009	−0.0534	−0.0037	−0.1095	−0.0020	−0.1027
*Listage*								
	(−0.21)	(−0.91)	(−0.10)	(−0.86)	(−0.24)	(−0.91)	(−0.13)	(−0.87)
	0.6207***	0.7107**	0.6504***	0.7395***	1.1687***	1.3542**	1.2243***	1.4078**
*Constant*								
	(9.98)	(2.59)	(9.78)	(2.62)	(9.90)	(2.59)	(9.70)	(2.61)
Observations	9703	524	9703	524	9703	524	9703	524
Industry	Yes	Yes	Yes	Yes	Yes	Yes	Yes	Yes
Year	Yes	Yes	Yes	Yes	Yes	Yes	Yes	Yes
*R* ^2^	0.1131	0.1881	0.1137	0.1865	0.1144	0.1870	0.1149	0.1855

***, **, and * represent passing the test at the significance levels of 1%, 5%, and 10%, respectively; the t-value has been robustly corrected during the statistical test.

In Table 7, Column (1) and Column (2) illustrate that when a manager is male, the coefficient between *PCN_Strength* and *Risk*_*T*1_*i*,*t*_ is 0.0865, significant at the 1% level. In the group of female managers, the coefficient of *PCN_Strength* and *Risk*_*T*1_*i*,*t*_ is not significant. Column (3) and Column (4) illustrate that the coefficient between *PCN_Density* and *Risk*_*T*1_*i*,*t*_ is 0.0505, significant at the 1% level. In the group of female managers, the coefficient of *PCN_Density* and *Risk*_*T*1_*i*,*t*_ is not significant. When *Risk*_*T*2_*i*,*t*_ is deployed to measure corporate risk-taking, the regression results are consistent with the analysis of *Risk*_*T*1_*i*,*t*_. It can be seen that the focal firms with male managers will be more influenced by perceived competition networks than those with female manager. These empirical results demonstrate that this personal trait can promote male managers to make more risky decisions, especially facing competition pressure from peer firms.

## Conclusion and Recommendations

### Conclusion

Corporate risk-taking is an important tool of risk management, and is also a key factor in determining financial performance and development capacity. The factors of corporate risk-taking mainly come from two dimensions, including the macro level and micro level. In the macro factors, industrial and national development strategies will change the level of corporate risk-taking, and these are uncertain factors. In the micro factors, financial features and manager characteristics can have a direct impact on corporate risk-taking, while these factors may also reflect the principal-agent problem. In the decision-making process of managers, corporate risk-taking would be influenced by the behaviors of other firms, indicating that the interaction between different firms may be a potential factor in affecting the risk bearing level of firms. Therefore, from the perspective of social network, exploring the impact of social interactions between firms on corporate risk-taking can better explain the motivation of corporate behaviors and business strategies.

Based on enterprise network, a special kind of network based on the perceived competition pressure of managers is proposed in this paper, named perceived competition network. Considering the nature of dynamic competition, we expand the measurement of competition in Li et al. (2013), and use the method of textual analysis to measure the semantic similarity of information disclosure among firms, and constructs the hidden inter-firm network for each focal firm. Under this hidden network, there is a close relationship between focal firm and its peer firms. In order to test whether competitive environment can influence corporate risk-taking, we discuss the impact of the characteristics of network on the risk bearing level of focal firm. The strength of perceived competition can promote corporate risk-taking, as well as the density of network. The perception of competition pressure could force managers to perform well, and this process may mitigate the agency conflicts by promoting corporate risk-taking. In terms of peer firms’ behaviors, the spillover effect of corporate risk-taking in perceived competition network can demonstrate that the risk bearing level of peer firms has become the learning motivation of focal firm, which can change the risk preference of managers. In terms of managers’ personal traits, the interactions between young managers or male managers have a stronger impact on corporate risk-taking. An alternative explanation may be that the age and gender of managers can strengthen their perceptions of competition pressure, and this will force managers to pay more attention to perceptible peer firms.

During exploring the factors of corporate risk-taking, we focus on the managers’ perceptions of competition pressure, and use the theoretical analysis and empirical analysis to verify the impact of perceived competition network on corporate risk-taking. Our findings can demonstrate that the social interactions between firms in perceived competition network would influence the decision-making of focal firm. The peer firms in this network can be seen as the relatively important perceptible peers, and the identification of these firms will be of great significance for the long-term development of firms.

### Recommendations and Limitations

According to the theoretical analysis and empirical analysis, there is a significant association between competition and corporate risk-taking, which can be explained by the risk preference of managers. These findings can help some firms in highly competitive environment to design appropriate development strategies, and enhance the sustainable ability of such firms. This paper may have the following implications:

First, the perceived pressure of managers can help them to identify some important peer firms. Considering the nature of competition, the similar goals of different firms can be reflected in the descriptive text of annual reports, which would be seen as the main ideas of managers. Using the semantic similarity of non-financial information disclosure, managers can pay constant attention to relative important peer firms, especially in different industries, and this will also represent the economic links between such firms.

Second, the risk bearing level of firms will be influenced by competitive environment. When managers face higher competition, they will work more on enterprise performance and focus on reducing managerial slack. For some firms in highly competitive environment, their managers need to learn from the decision-making of peer firms, and this interaction can help them to design some appropriate strategies for achieving the goals of long-term development.

This paper has several limitations, and needs to be improved in the future research. In terms of research sample, we choose A-share listed firms in the Chinese market, and remove the sample of other listed firms in Small and Medium-sized Enterprise Board and Growth Enterprises Market. The limitation of sample can not allow us to measure the degree of new entry threats, and there are some inaccuracies in the measurement of perceived competition pressure faced by managers. In terms of research period, the data of listed firms after 2018 is not added in empirical analysis, and the exploring process of factors of corporate risk-taking will not consider the external shocks, such as the impact of COVID-19. In the future research, we will expand the research sample and period, and consider the impact of new entry threats and external shocks on corporate risk-taking. Furthermore, the whole perceived competition network will be constructed based on China’s listed firms, and the characteristics of whole network will be the motivation of our future research.

## Data Availability Statement

The data sources of this article are the Chinese Research Data Services Platform Database (https://www.cnrds.com/) and the China Stock Market and Accounting Research (CSMAR) Database (https://www.gtarsc.com/).

## Author Contributions

FC and WW contributed to the conceptualization. FL contributed to the methodology and visualization. YG contributed to the software. JX contributed to the formal analysis. FC and JX contributed to the writing—original draft preparation. WW and YG contributed to the writing—review and editing. All authors have read and agreed to the published version of the manuscript.

## Conflict of Interest

The authors declare that the research was conducted in the absence of any commercial or financial relationships that could be construed as a potential conflict of interest.

## Publisher’s Note

All claims expressed in this article are solely those of the authors and do not necessarily represent those of their affiliated organizations, or those of the publisher, the editors and the reviewers. Any product that may be evaluated in this article, or claim that may be made by its manufacturer, is not guaranteed or endorsed by the publisher.
